# Gut microbiota metabolites mediate the interplay between childhood maltreatment and psychopathology in patients with eating disorders

**DOI:** 10.1038/s41598-023-38665-x

**Published:** 2023-07-20

**Authors:** Giovanni Castellini, Emanuele Cassioli, Francesco Vitali, Eleonora Rossi, Cristiano Dani, Giulia Melani, Dario Flaccomio, Martina D’Andria, Mariela Mejia Monroy, Andrea Galli, Duccio Cavalieri, Valdo Ricca, Gian Luca Bartolucci, Carlotta De Filippo

**Affiliations:** 1 Department of Health Sciences, University of Florence, Florence, Italy; 2 Institute of Agricultural Biology and Biotechnology, National Research Council, Pisa, Italy; 3 Gastroenterology Research Unit, Department of Experimental and Clinical Biomedical Sciences “Mario Serio”, University of Florence, Florence, Italy; 4 Department of Biology, University of Florence, Florence, Italy; 5 Department of Neurosciences, Psychology, Drug Research and Child Health, University of Florence, Florence, Italy

**Keywords:** Biochemistry, Microbiology, Neuroscience, Psychology, Gastroenterology, Medical research, Risk factors

## Abstract

Eating disorders (EDs) are syndromes with a multifactorial etiopathogenesis, involving childhood traumatic experiences, as well as biological factors. Human microbiome has been hypothesised to play a fundamental role, impacting on emotion regulation, as well as with eating behaviours through its metabolites such as short chain fatty acids (SCFAs). The present study investigated the interactions between psychopathology of EDs, the gut microbiome and SCFAs resulting from bacterial community metabolic activities in a population of 47 patients with Anorexia Nervosa, Bulimia Nervosa, and Binge Eating Disorder and in healthy controls (HCs). Bacterial gut microbiota composition differences were found between subjects with EDs and HCs, especially in association with different pathological behaviours (binge-purge vs restricting). A mediation model of early trauma and ED-specific psychopathology linked reduction of microbial diversity to a typical microbiota-derived metabolite such as butyric acid. A possible interpretation for this model might be that childhood trauma represents a risk factor for gut dysbiosis and for a stable modification of mechanisms responsible for SCFAs production, and that this dysfunctional community is inherited in the passage from childhood to adulthood. These findings might open the way to novel interventions of butyric acid-like compounds as well as faecal transplant.

## Introduction

Eating disorders (EDs)—including Anorexia Nervosa (AN), Bulimia Nervosa (BN) and Binge Eating Disorder (BED) represent a public health concern^1,2^, due to the high medical and psychiatric comorbidities^3^, the frequent hospitalisation, the high rate of chronicity (40%) and mortality (5–10%)^4^. EDs are syndromes with a multifactorial etiopathogenesis, involving childhood traumatic experiences, as well as social, psychological, and biological factors^5–7^. However, the lack of integration between clinical and biological data might represent limitations of the actual treatment models^4^, accounting for the scarce efficacy of pharmacological treatments, as compared to other psychiatric conditions^8^. Within biological factors, microbiota-gut-brain-axis (i.e., mostly bacteria and fungi stably colonising the gut, with their genes, proteins and metabolites affecting host physiology^9^, has been recently suggested in the pathogenesis and maintenance of EDs^10,11^, considering its crucial role in the homeostasis of the host system^12,13^. Indeed, the signals from the gastrointestinal tract can influence several brain functions and behaviours, including food-seeking and eating behaviours. Communication in this system does not only depend on neuronal signals, but also on endocrine molecules (hormones and gut peptides), and gut microbiota derived metabolites, acting together to regulate host physiology^12^. Some microorganisms (e.g. *Clostridium perfringens, Escherichia coli, Lactobacillus, Bifidobacterium*) have been shown to synthesise and respond to the main neurochemical compounds implicated in psychopathology (i.e. DA, 5-HT, GABA, etc.)^14,15^ or to metabolise their precursors such as tryptophan, glutamic acid, tyrosine, and phenylalanine. Metabolites produced by the gut microbiota—such as short chain fatty acids (SCFAs), can modulate eating behaviours^16,17^, attenuating for example ghrelin receptor signalling^18^ or inducing anorectic hormones^17^. Furthermore, the modulation of GABAergic, serotoninergic, and dopaminergic neurotransmission induced by SCFAs^19–23^, might influence other psychopathological dimensions such as anxiety or depression, which are involved in the maintenance of EDs psychopathology itself.

However, only preliminary findings suggest a role of the gut microbiota in the aetiology and progression of EDs, and no conclusive study has been performed integrating biological data into the psychopathological models on which are based the available treatments. Indeed, a considerable heterogeneity should be noticed in the evidence reported by scientific literature regarding gut microbiota, SCFAs and EDs^22^, with controversial results about the presence of alterations in the gut microbiota of patients with AN^24,25^.

Several different methodological explanations should be provided to explain heterogeneity in the research on the role of microbiota alterations in EDs (i.e. methodological heterogeneity from stool sample conservation, DNA extraction to 16S rRNA NGS procedure; heterogeneity of patients included in terms of diet, BMI, origins). However non conclusive results might be due also to the well-known limits of the comparisons based on diagnostic categorization. They are often inconsistent in the field of EDs, considering that diagnoses are based on state-dependent symptoms (i.e. eating behaviours and anthropometric data)^26,27^, even though previous studies found limited effect of state-dependent weight restoration on gut microbiota alterations^28^ On the contrary, analyses of trait features and predisposing factors for EDs could be more useful to provide significant associations with gut-brain-axis functioning. For example, it is well known that adverse experiences during childhood (especially abuse and neglect), are predisposing factors for EDs^29^, and might individuate a sub-population of patients with specific psychopathological features^30^ and outcome^7^, as well as distinct biological underpinnings^31^. Several evidence^31^ seem to indicate that EDs with history of childhood trauma report dysregulated hypothalamic pituitary adrenal (HPA) axis function in adulthood, which might account for both psychiatric comorbidity^7^ due to severe emotion dysregulation, and medical comorbidity due to impaired immune function^32–36^. Recently, it has been suggested that the relationship between childhood trauma and alterations in HPA might be mediated by the gut microbiota, and its metabolites^37^.

The present study attempted to overcome the limitation of the studies conducted so far, proposing an integrated explicative model for psychopathology associated with EDs, including the presence of childhood adverse experiences, gut microbiota composition and its metabolites (SCFAs). Thus, the investigation was aimed at evaluating the association of the gut microbiota composition and its metabolites (SCFAs) with pathological behaviours, and psychopathology. In particular, the present study tested whether SCFAs represented a possible mediator of the relationship between adverse early life experiences (abuse and neglect) and psychopathology in patients with EDs. Then, the study evaluated whether changes in psychopathology would be related to the bacterial community and especially assessing the relative metabolic activity which has been shown to be dominant with respect to the taxonomic distribution as observed in the work of the Human Microbiome Project Consortiumthat observed that in contrast to taxa, few pathways were highly variable among subjects indicating a functional redundancy define as “core gut microbiota”^38^ the metabolic activity (SCFAs) of the bacterial community, in relation to its diversity and composition. Compared with most of the previous studies in this field which were focused on AN, the present investigation involved all the three main diagnoses, namely AN, BN, and BED, according to a trans-nosographic approach to EDs psychopathology.

## Results

### Clinical and psychopathological characteristics

We analysed the clinical and psychopathological characteristics in a cohort of ED-affected subjects (Table 1). The average age was 27.27 ± 2.47 years in the HC group and 24.14 ± 7.09 in the patients’ group, while the average level of education was 17.85 ± 2.35 and 15.82 ± 2.87, respectively. A total of 10 patients were receiving antidepressant therapy at the time of assessment, while 4 were taking benzodiazepine anxiolytics. Through clinical evaluation and administration of the CTQ, a total of 22 (46.8%) patients reported adverse childhood experiences.

**Table 1:** Clinical and psychopathological characteristics of the sample, divided by groups.

	AN (n = 21)	BN (n = 17)	BED (n = 9)	HCs (n = 28)	F
BMI (kg/m^2^)	15.96 ± 1.37^†‡§^	25.27 ± 8.50^‡§^	35.53 ± 8.45^§^	20.73 ± 1.80	29.95***
SCL-90-R GSI	1.26 ± 0.71^§^	1.38 ± 0.83^§^	1.54 ± 1.09^§^	0.43 ± 0.38	9.41***
EDE-Q dietary restraint	2.77 ± 2.20^§^	2.83 ± 1.54^§^	1.62 ± 1.91	0.48 ± 0.91	10.27***
EDE-Q eating concerns	2.07 ± 1.57^§^	3.21 ± 1.60^§^	3.45 ± 1.80^§^	0.42 ± 0.59	14.39***
EDE-Q weight concerns	2.44 ± 1.81^‡§^	3.59 ± 1.86^§^	4.17 ± 1.02^§^	0.72 ± 0.97	13.01***
EDE-Q shape concerns	2.82 ± 1.79^‡§^	3.83 ± 2.02^§^	4.67 ± 1.23^§^	1.23 ± 1.34	8.78***
EDE-Q total score	2.52 ± 1.72^§^	3.36 ± 1.46^§^	3.48 ± 1.29^§^	0.71 ± 0.90	13.88***
Binge eating frequency	3.93 ± 9.79^†^	12.58 ± 12.06^§^	11.88 ± 9.61^§^	0.15 ± 0.46	6.02**
Compensatory exercise frequency	2.92 ± 8.15	7.33 ± 11.12^§^	0.00 ± 0.00	0.08 ± 0.39	3.83*
Self-induced vomiting frequency	2.00 ± 5.18†	8.85 ± 12.21^‡§^	0.00 ± 0.00	0.00 ± 0.00	6.69***
CTQ emotional neglect	10.53 ± 5.17‡	10.53 ± 4.91	15.50 ± 4.57^§^	8.62 ± 3.10	2.94*
CTQ emotional abuse	7.76 ± 4.52	8.67 ± 5.59	10.75 ± 4.98	5.81 ± 1.47	2.26
CTQ sexual abuse	6.00 ± 2.35	6.53 ± 3.31	9.12 ± 5.69	5.19 ± 0.63	1.43
CTQ physical neglect	6.24 ± 2.02	7.27 ± 3.47	8.50 ± 3.96	5.58 ± 1.14	1.44
CTQ physical abuse	5.24 ± 0.83	6.93 ± 5.08	6.75 ± 4.30	5.23 ± 0.86	0.72
CTQ total score	42.94 ± 14.25^‡§^	48.40 ± 18.84^§^	59.75 ± 16.93^§^	37.00 ± 6.23	2.90*
STAI state anxiety	49.06 ± 16.02^§^	50.81 ± 16.49^§^	52.88 ± 13.28^§^	37.62 ± 10.14	4.07*
STAI trait anxiety	51.94 ± 13.00^§^	51.25 ± 17.26	55.50 ± 11.07^§^	41.12 ± 9.45	3.75*
EES anger	9.72 ± 10.50^‡^	18.77 ± 15.85^§^	28.38 ± 8.77^§^	6.73 ± 8.28	4.74**
EES anxiety	8.56 ± 6.53^‡^	14.38 ± 12.97^§^	22.00 ± 6.63^§^	5.62 ± 6.75	4.29**
EES depression	5.17 ± 4.09^†‡^	10.54 ± 7.01^§^	13.00 ± 6.07^§^	4.88 ± 4.41	4.29**
EES total score	23.44 ± 20.24^‡^	43.69 ± 34.38^§^	63.38 ± 19.67^§^	17.23 ± 18.32	4.87**

*BMI* body mass index, *SCL-90-R GSI* symptom checklist 90 revised global severity index, *EDE-Q* eating disorders examination questionnaire, *CTQ* childhood trauma questionnaire, *STAI* state-trait anxiety inventory, *EES* emotional eating scale.

BMI-adjusted comparisons are reported, with post hoc indicated using the following symbols: ^†^ (significantly different than BN), ^‡^ (significantly different than BED), ^§^ (significantly different than HCs); p-values: *p < 0.05, **p < 0.01, ***p < 0.001.

Patients reported higher levels of general and ED-specific psychopathology, early trauma, anxiety, and ED-related behaviour (Table 1).

### Association of psychopathological variables and faecal SCFAs: role of butyric acid in trait anxiety prediction

**Figure 1:**
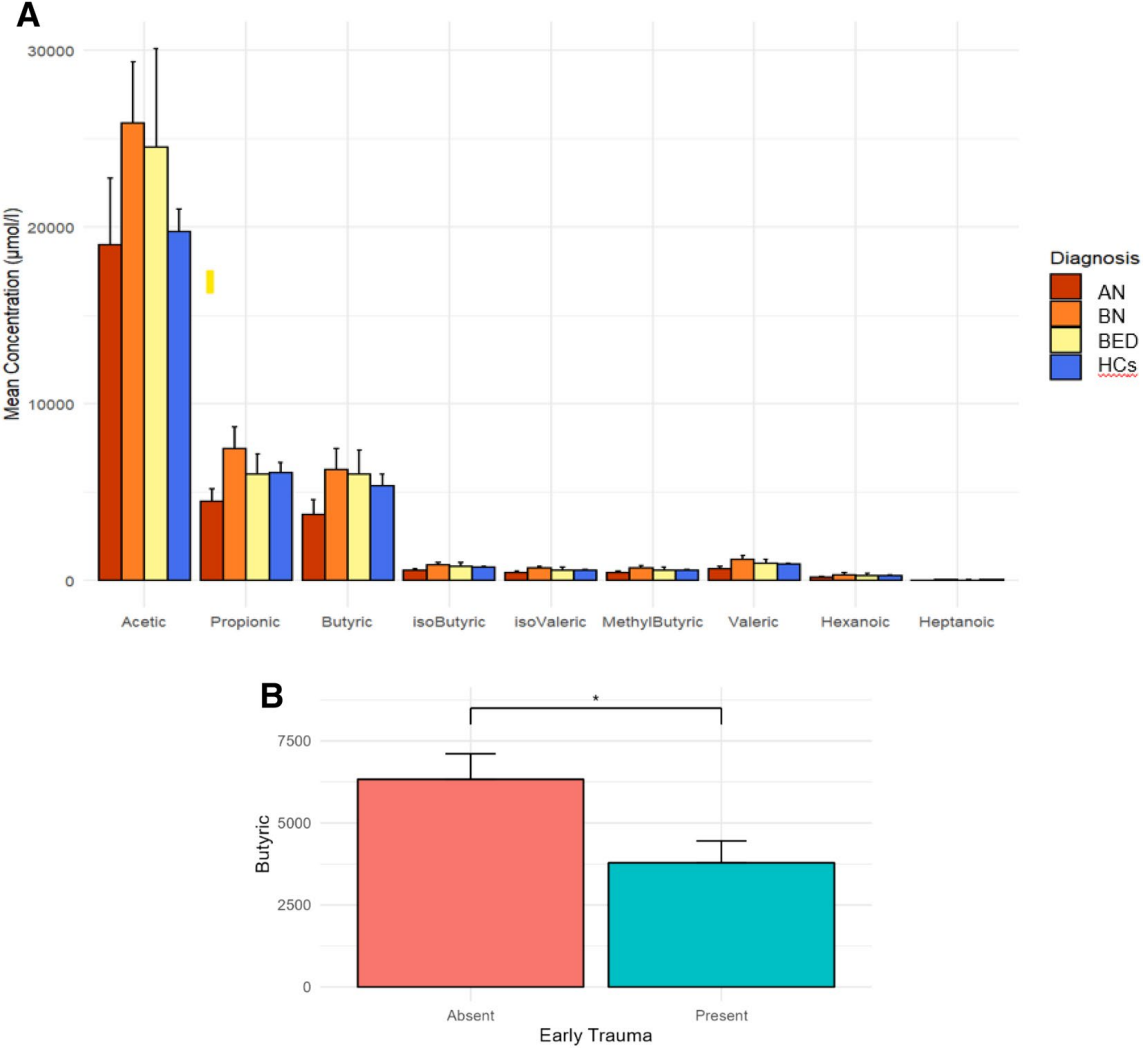
Fatty acids concentrations in patients (divided by diagnosis) and HCs (panel (**A**)), and in patients with and without early trauma (panel (**B**)). Error bars illustrate standard errors. AN, anorexia nervosa; BN, bulimia nervosa; BED, binge eating disorder; HCs, healthy controls. When dividing patients in terms of high (greater than the cutoff value of 35) vs low CTQ scores, patients in the first group reported significantly reduced butyric acid as compared to the latter group.

We analysed SCFAs and MCFAs in stool samples of all ED subjects to try to understand how much these metabolites related to the intestinal microbiota could represent biomarkers of alteration of intestinal homeostasis, and their association with the psychopathological variables in these patients in whom there is no longer a physiologically correct diet. Fatty acids concentrations in different groups are illustrated in Fig. 1. No significant differences were found among groups.

No significant associations were found between clinical variables and SCFAs in HCs. Among patients, BMI was not significantly associated with fatty acids concentrations, except for valeric acid (β = 0.38, p = 0.003). BMI-adjusted psychometric associations of fatty acids are reported in Table 2. Childhood emotional neglect and abuse were negatively associated with shorter SCFAs, and CTQ Total Score was associated with propionic and butyric acids (Table 2). Higher levels of trait anxiety were significantly associated with lower concentrations of all fatty acids except for hexanoic and heptanoic acids (Table 2). Anger-related emotional eating (EES Anger) was negatively associated with butyric, isobutyric, isovaleric, 2-methylbutyric, valeric and hexanoic acids, whereas EES Depression was negatively associated with hexanoic acid (Table 2). No association was found for general psychopathology, whereas total ED-specific psychopathology was associated with lower levels of isovaleric and 2-methylbutyric acids (Table 2).

**Table 2:** Associations between fatty acid concentrations and psychometric measures, investigated in the group of patients (n = 47) through BMI-adjusted multiple regression analyses.

	Acetic acid	Propionic acid	Butyric acid	isoButyric acid	isovaleric acid	2-MethylButyric acid	Valeric acid	Hexanoic acid	Heptanoic acid
SCL-90-R GSI	− 0.14	− 0.20	− 0.24	− 0.26	− 0.28	− 0.31	− 0.27	− 0.22	− 0.07
EDE-Q dietary restraint	− 0.01	− 0.07	− 0.02	− 0.14	− 0.18	− 0.19	0.07	− 0.07	− 0.01
EDE-Q eating concerns	− 0.08	− 0.13	− 0.09	− 0.19	− 0.22	− 0.23	− 0.11	− 0.11	− 0.01
EDE-Q weight concerns	− 0.11	− 0.14	− 0.28	− 0.33*	− 0.37*	− 0.39*	− 0.34*	− 0.37*	− 0.19
EDE-Q shape concerns	− 0.13	− 0.21	− 0.33*	− 0.36*	− 0.40*	− 0.41**	− 0.31	− 0.33*	− 0.13
EDE-Q total score	− 0.10	− 0.16	− 0.21	− 0.29	− 0.34*	− 0.35*	− 0.20	− 0.25	− 0.10
Binge eating frequency	− 0.06	0.12	0.06	0.04	0.02	0.06	0.04	− 0.29	− 0.34
Compensatory exercise frequency	0.00	0.17	− 0.02	− 0.22	− 0.24	− 0.23	− 0.13	− 0.15	− 0.20
Self-induced vomiting frequency	− 0.12	− 0.21	− 0.23	− 0.15	− 0.12	− 0.13	− 0.13	− 0.17	− 0.06
CTQ total score	− 0.23	− 0.32*	− 0.33*	− 0.23	− 0.20	− 0.23	− 0.32	− 0.23	− 0.18
STAI state anxiety	− 0.21	− 0.21	− 0.33	− 0.24	− 0.21	− 0.27	− 0.29	− 0.16	0.04
STAI trait anxiety	− 0.44**	− 0.47**	− 0.55***	− 0.40*	− 0.37*	− 0.41*	− 0.52**	− 0.27	− 0.06
EES anger	− 0.14	− 0.23	− 0.33*	− 0.36*	− 0.36*	− 0.36*	− 0.40*	− 0.35*	− 0.23
EES anxiety	− 0.06	− 0.14	− 0.26	− 0.28	− 0.29	− 0.30	− 0.32	− 0.28	− 0.16
EES depression	− 0.13	− 0.17	− 0.30	− 0.16	− 0.14	− 0.15	− 0.31	− 0.36*	− 0.26
EES total score	− 0.12	− 0.20	− 0.31	− 0.30	− 0.30	− 0.31	− 0.36*	− 0.34*	− 0.22

*SCL-90-R GSI* symptom checklist 90 revised global severity index, *EDE-Q* eating disorders examination questionnaire, *CTQ* childhood trauma questionnaire, *STAI* state-trait anxiety inventory, *EES* emotional eating scale.

Standardised regression coefficients (β) are reported; p-values: *p < 0.05, **p < 0.01, ***p < 0.001.

Considering possible confounders, no significant association was found between SCFAs and general psychopathology, depression as measured by SCL-90, diet (daily mean calorie consumption), or inflammation (VES scores) (data not shown).

**Figure 2:**
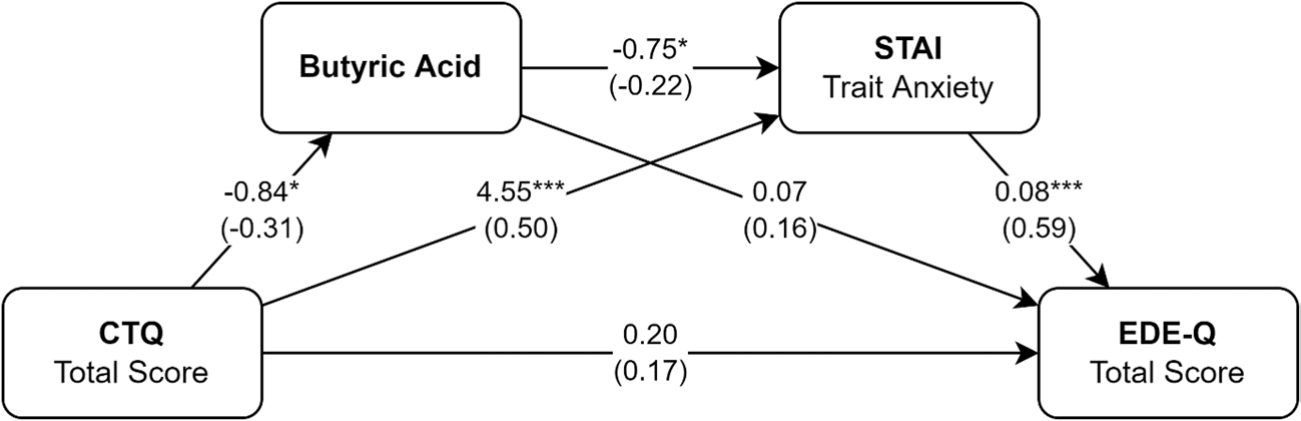
Serial mediation model including butyric acid and trait anxiety as mediators for the association between early trauma and ED psychopathology. Unstandardized coefficients are reported, with standardised coefficients in parenthesis. All analyses were BMI-adjusted. Butyric concentrations were divided by a factor of 10^3^, whereas CTQ scores were divided by 10: regression coefficients should be interpreted accordingly; p-values: *p < 0.05, **p < 0.01, ***p < 0.001. *CTQ* childhood trauma questionnaire, *EDE-Q* eating disorders examination questionnaire, *STAI* state-trait anxiety inventory.

As shown in Fig. 2, we propose a mediation model linking early trauma and ED-specific psychopathology through butyric acid and trait anxiety in series. Butyric acid significantly predicted higher levels of trait anxiety, which in turn was associated with EDE-Q Total Score; moreover, both mediators (butyric acid and trait anxiety) were associated with early trauma (Fig. 1). Consequently, all the criteria for conducting a serial mediation analysis were met. This analysis confirmed the existence of a serial indirect effect of early trauma on ED psychopathology, mediated by lower levels of butyric acid which in turn correlated with higher trait anxiety (b = 0.05, 95% CI [0.01, 0.18]). A second mediation was found, through higher STAI Trait Anxiety scores only (b = 0.35, 95% CI [0.16, 0.63]). The total effect of CTQ Total Score on EDE-Q Total Score was statistically significant (b = 0.54, p < 0.001). The abovementioned analyses were all BMI adjusted.

### Microbiota profiles and psychopathology

We characterised the bacterial faecal microbiota associated with EDs by means of high-throughput sequencing of the V3-V4 region of the 16S rDNA gene. We quantified the bacterial richness within each sample (alpha-diversity) of the four groups, AN (n = 21), BN (n = 17), BED (n = 9), and 28 healthy controls (hereinafter termed HCs). Three different alpha diversity estimators were used, namely the Richness, Pielou’s Evenness, and Shannon’s index. The bacterial gut microbiota profile of EDs subjects was significantly less diverse compared to that of HCs (p < 0.005, Wilcoxon rank-sum test) with all the three estimators used. To better understand the existence of a faecal microbial profile associated with the eating behaviour of the patients enrolled in the study, as a first analysis, we aimed at evaluating the most useful factor to stratify samples in relation to the gut microbiota. We performed permutational ANOVA analysis to test the relation between the Bray–Curtis distance matrix of the gut microbiota communities, and several stratification factors. All the stratification resulted significant and independent from BMI (as indicated by the not significance of the interaction term), suggesting that gut microbiota composition is differing among groups, whatever grouping was used. As expected, detailing the patients sub-grouping based on diagnosis improved the distinction between microbial communities (higher R^2^, with a non-significant BMI factor effect), in both cases (stratification Group 2 and Diagnosis in Table 3) a further inspection with pairwise-PERMANOVA indicated that the HCs group was always different respect to all other groups, with more differences between BED and AN-r.

**Table 3:** Results of PERMANOVA test to check the association between different samples stratification and microbial community.

Stratification	Df	F. model	R2	p	BMI factor p	BMI interaction (p)	Notes
Group (HCs, P)	1	3.238	0.057	0.0001	0.0016	ns	
Group 2 (HCs, AN, BED, BN)	3	1.920	0.102	0.0001	ns	ns	Pairwise comparisons HCs different from all other, no difference between diagnosis
Diagnosis (HCs, AN-bp, AN-r, BED, BN)	4	1.796	0.126	0.0001	ns	ns	Pairwise comparisons HCs different from all other, no difference between diagnosis. BED vs AN-r trending
Diagnosis 2 (HCs, binge-purging, restricting)	2	2.709	0.09	0.0001	ns	ns	Pairwise comparison all group different

For every test, a different sample grouping (reported in the “stratification” column) is tested against the Bray–Curtis distance matrix of the bacterial community, correcting for BMI (i.e. using an interaction term in the PERMANOVA model).

*AN* anorexia nervosa, *AN-bp* anorexia nervosa binge-purging, *AN-r* anorexia nervosa restricting, *BN* bulimia nervosa, *BED* binge eating disorder, *HCs* healthy controls, *BMI* body mass index.

Finally, partially addressed by this last observation, we tested a further type of sample grouping. Based on their overall behaviour we classified patients in “restricting” (i.e., AN-r; patients with a behaviour characterised by dietary restriction with/without compensatory physical activity) and “Binge-Purge” (i.e., BN, BED and AN-bp; patients with a behaviour characterised by binge-eating with/without purging conducts). At testing with PERMANOVA, this grouping performed comparably to the previous one (R^2^ = 0.09, p-value = 0.0001, no interaction with BMI), nevertheless it performed arguably better than the previous at post-hoc comparison between levels. In this case, in fact, pairwise-PERMANOVA test indicated a significant difference between all the pairwise comparison (HCs vs binge-purge; HCs vs. restricting; binge-purge vs. restricting), representing the tested stratification able to differentiate the faecal microbial communities between control and ED patients, considering different stratification of patients.

Considering the model reported in Fig. 2, in which butyric acid and trait anxiety mediated the association between early trauma and ED psychopathology, it was used the same permutational ANOVA framework to assess if those variables also had an influence on the microbial communities (Table 4). All the tests were performed separately to evaluate which variable had a significant effect, and to evaluate if this effect had any interactions with the BMI. The microbial community was significantly related to the butyric acid, the STAI (Trait Anxiety), and the Total EDE score. In every case, there was also a statistically significant relation to the BMI, also if the interaction was significant only for the Total EDE score.

**Table 4:** Results of PERMANOVA test to check the association between variables included in the serial mediation model, and microbial communities.

	F. model	R2	p	BMI factor p	BMI interaction p
Butyric Acid	1.429	0.027	0.026	0.020	ns
STAI_T	1.368	0.025	0.042	0.001	ns
CTQ_Tot	1.104	0.021	ns	0.015	ns
EDE-Q_Tot	2.333	0.042	0.000	0.001	0.028

Every variable is tested against the Bray–Curtis distance matrix of the bacterial community, correcting for BMI (i.e., using an interaction term in the PERMANOVA model).

*STAI_T* state-trait anxiety inventory, *CTQ* childhood trauma questionnaire, *EDE-Q* eating disorders examination questionnaire.

**Figure 3:**
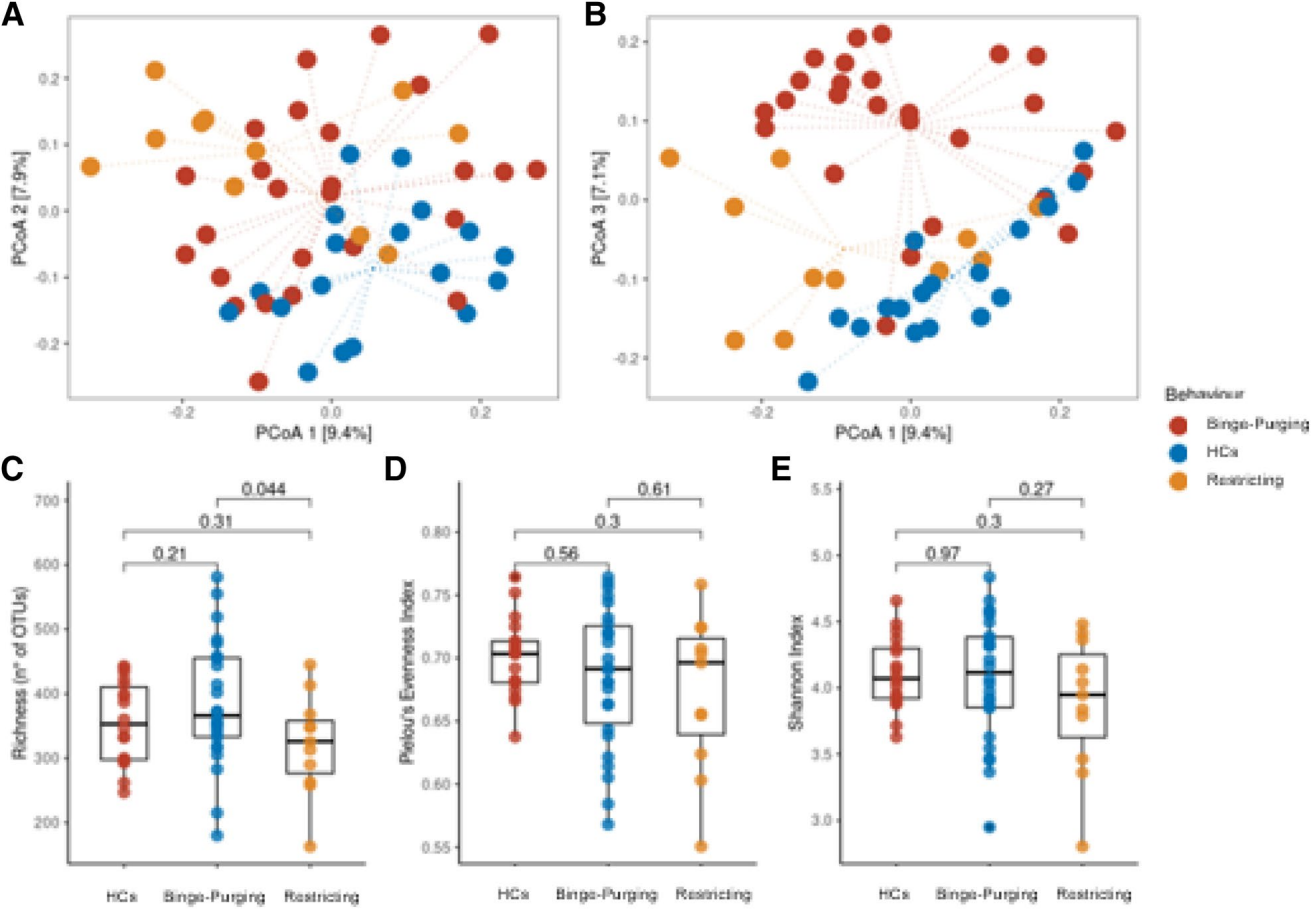
Diversity analysis based on the behaviour classification of ED patients, respect to HCs samples. Beta diversity analysis (**A,B**) was performed with PCoA ordination based on the Bray–Curtis dissimilarity measure, plotting the 1st and 2nd (**A**) or the 1st and 3rd (**B**) ordination axis. Dotted lines connect each point to the centroid of its sample group defined by the Behaviour variable. Alpha diversity analysis: boxplot reporting Richness (**C**) Pielou’s Evenness (**D**) and Shannon’s index (**E**) in the groups. Differences were tested with Wilcoxon and resulting p-value was reported as annotation on the plot. HCs, healthy controls.

In addition, ordination analysis and alpha diversity evaluation was also performed considering the behaviour classification (Fig. 3). Considering the associations observed between faecal microbiota composition with both pathological behaviours (restricting vs binge-purging) and some psychopathology indices of the mediation model (STAI_T, EDE_Tot, and butyric acid), sample diversity was additionally evaluated using alpha and beta diversity analysis in consideration of these variables (Fig. 3).

Interestingly the 3rd ordination axis was able to differentiate both between binge-purging group (on the upper part of the ordination plot) and the other samples (Fig. 3B), and between restricting patients and healthy controls. Interestingly, this clusterization also corresponds to a difference in EDE and STAI-T measure (higher values in binge subjects, Fig. 3). Alpha diversity (Fig. 3C–E) did not show significant differences between sample groups, except for richness which resulted significantly lower in restricting patients with respect to binge-purging patients.

**Figure 4:**
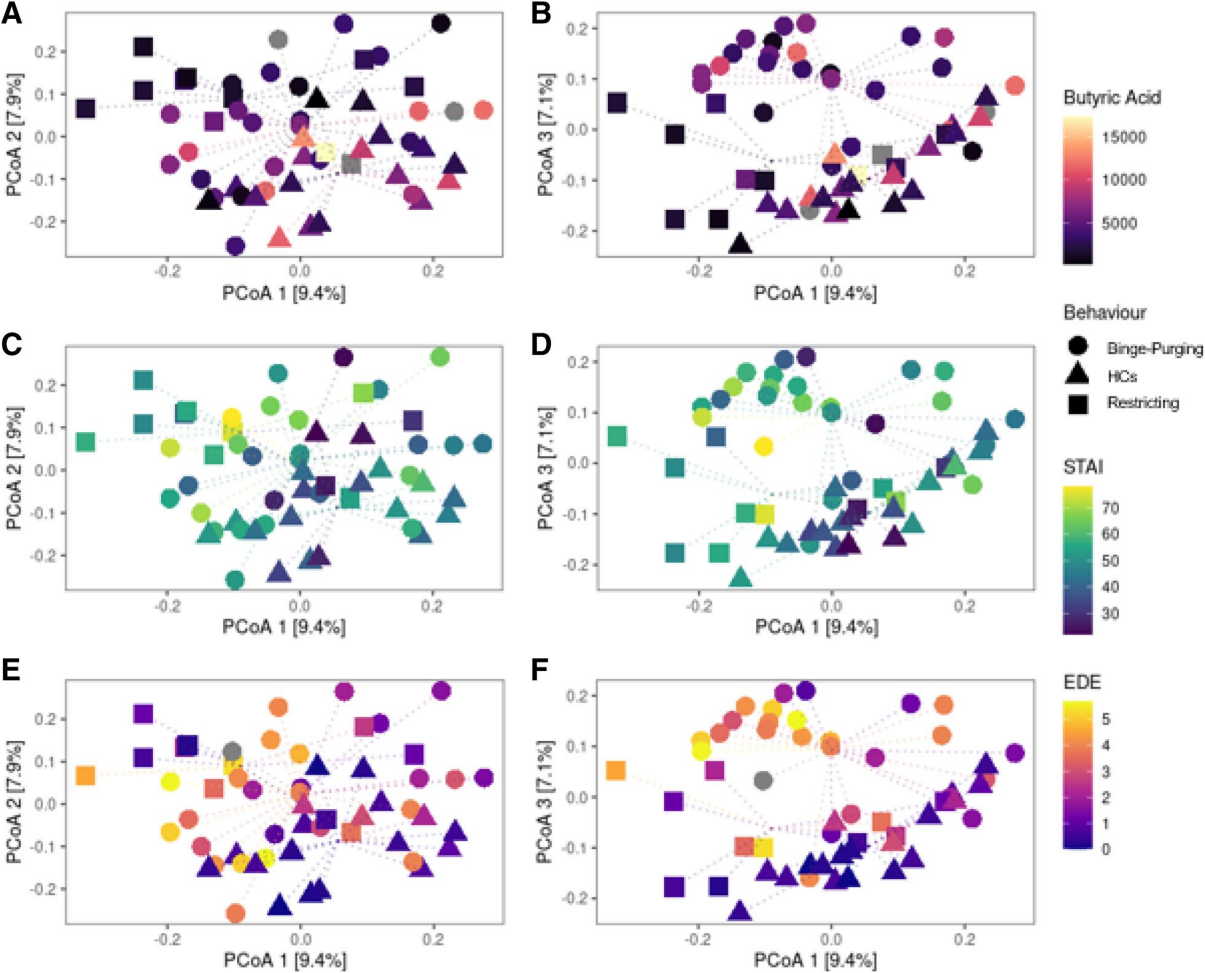
Diversity analysis based on the behaviour classification of ED patients, respect to HCs samples. Beta diversity analysis was performed with PCoA ordination based on the Bray–Curtis dissimilarity measure, plotting the 1st and 2nd (**A,C,E**) or the 1st and 3rd (**B,D,F**) ordination axis. Dotted lines connect each point to the centroid of its sample group defined by the Behaviour variable (shape). Points are coloured on the basis of variables in the serial mediation model which showed a significant relation to diversity in PERMANOVA analysis; the butyric acid (**A,B**), the STAI (**C,D**), and the EDE (**E,F**). HCs, healthy controls; *EDE* eating disorders examination questionnaire, *STAI* state-trait anxiety inventory.

**Figure 5:**
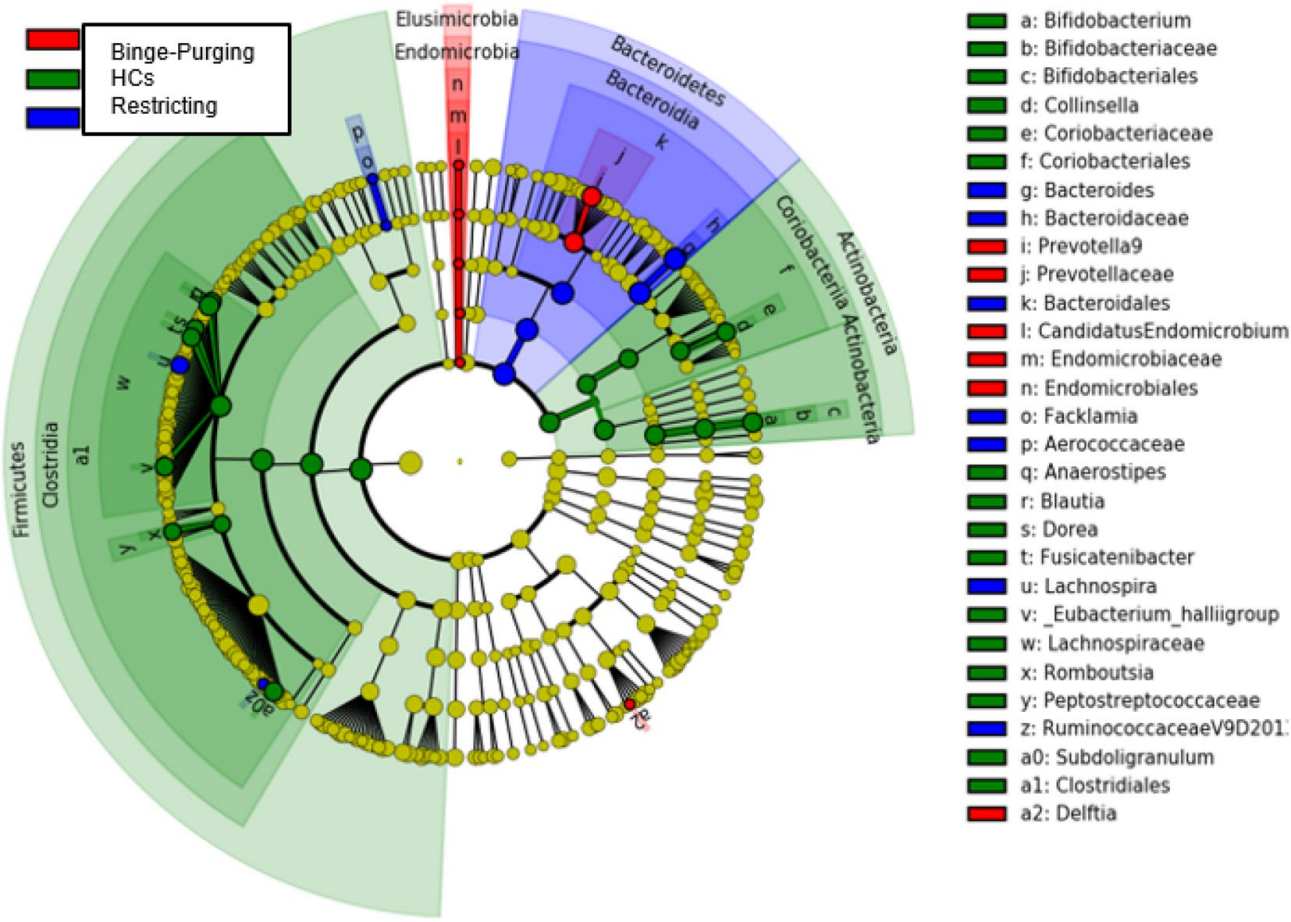
LEfSe analysis based on the behaviour classification of ED patients, respect to HCs samples. As a high number of putative markers were identified by the analysis (in *one-against-all* mode) we used a higher than usual LDA score threshold (LDA threshold = 3.5) to focus on the most differentiating markers. HCs, healthy controls.

Despite there are apparently moderate differences in alpha and beta diversity between HCs, restricting patients, and binge-purging patients, differential abundance analysis revealed a substantially different bacterial community (Fig. 4) between the groups. LEfSe analysis was adopted for the discovery of differentially abundant features of the bacterial community, restricting our evaluation to the most interesting by using a high LDA score threshold (LDA threshold = 3.5). Differences were obvious already at high taxonomic levels, with the Phyla of Actinobacteria and Firmicutes specific to healthy subjects’ gut microbiota profiles, while Bacteroidetes of the Restricted and Binge-Purge. At the Genus level (Fig. 5), binge-purging patients were characterised by the relative abundance of *Prevotella*, while restricting patients were characterised by *Bacteroides*, and by the Firmicutes phyla members such as *Facklamia* and *Lachnospira*. Respect to patients with EDs, healthy subjects had higher relative abundances of genera *Bifidobacterium* and *Collinsella* belonging from the Actinobacteria, as well as higher relative abundances of *Anaerostipes*, *Blautia*, *Dorea*, *Fusicatenibacter*, *Romboutsia*, *Subdoligranulum* and *Eubacterium hallii* group.

Three different clusters were identified in mode details (Table 5):

- Cluster 1 (a mixed cluster of 23 samples, 10 ED patients 44%, and 13 controls 56%) is the most abundant sample cluster. It is characterised by samples with lower-than-median values for almost all variables (except EDE-total). The almost total balance between control and patients with EDs, as well as the even distribution of patients in restricting and binge-purging behaviour.
- Cluster 2 (a mixed cluster of 9 samples, 5 ED patients and 4 control) has almost even composition among control and patients but it is mainly composed of patients reporting binge-purging (4 out of 5). It is the youngest cluster (median age 25), and it is characterised by higher than overall median values of BMI and EDE-total, but lower than overall median values of CTQ-total and STAI-T. Peculiar feature of this cluster, is the extremely high butyric acid content of the faeces, reaching a median value which is three times the one of the overall datasets.
- Cluster 3 is the second most abundant cluster. It is mainly composed of patients with EDs (16 out of 17) of which the majority is characterized by binge-purging behaviour (13 out of 16). Consequently, the ED patients in this cluster are characterised by higher than overall median values of all pathological indices, as BMI, Age, CTQ-total, STAI-T and EDE-total, but with lower than overall median quantity of butyric acid.

**Table 5:** Characteristics of samples in the three clusters of HCPC analysis.

Cluster	TOT	HCs	ED	Restricting	Binge-purging	BMI median	Age median	Butyric acid median	CTQ-total median	STAI-T median	EDE-Q total median
1	23	13	10	5	5	19.03 ± 2.72	27 ± 8.18	3121.84 ± 2007.37	36 ± 8.65	40 ± 10.39	0.62 ± 0.74
2	9	4	5	1	4	22 ± 9.35	25 ± 8.82	11,372.73 ± 2921.72	36 ± 8.37	41 ± 7.47	2.03 ± 1.36
3	17	1	16	3	13	24.2 ± 10.43	30 ± 17.39	3462.46 ± 2916.52	54 ± 16.07	58 ± 6.98	4.06 ± 1.27
Overall	49	18	31	9	22	19.81 ± 8.15	27 ± 12.74	3719.149 ± 3859.74	40 ± 14.44	48 ± 13.14	0.24 ± 1.73
ANOVA				Cluster		5.81**	2.70	37.13***	14.27***	30.20***	49.10***
				Behavior		3.91*	1.59	0.32	1.80	0.28	5.1 *
				Interaction		0.998	0.14	2.73*	1.40	1.46	1.6

*HCs* healthy controls, *ED* eating disorder, *BMI* body mass index, *CTQ* childhood trauma questionnaire, *STAI_T* state-trait anxiety inventory, *EDE-Q* eating disorders examination questionnaire.

Reported values are median ± standard deviation. ANOVA was used to test each variable respect to the cluster and behaviour factors, and their interaction; p-values: *p < 0.05, **p < 0.01, ***p < 0.001.

As expected, ANOVA analysis confirms that the cluster variable was always the most significant in explaining the variables of the mediation model, as well as the BMI, but was not significant with respect to age.

### Beyond patient’s diagnosis: data driven stratification of ED patients

Finally, having proved the existing connections between bacterial community diversity and the variables included in the mediation model, and having proved how the model was able to link early trauma and ED-specific psychopathology traits, with a typical gut microbiota-derived metabolite such as butyric acid, it was tested if a data-driven patients' classification was able to better recapture the connection, suggested by those results, between gut microbiota and psychopathology.

**Figure 6:**
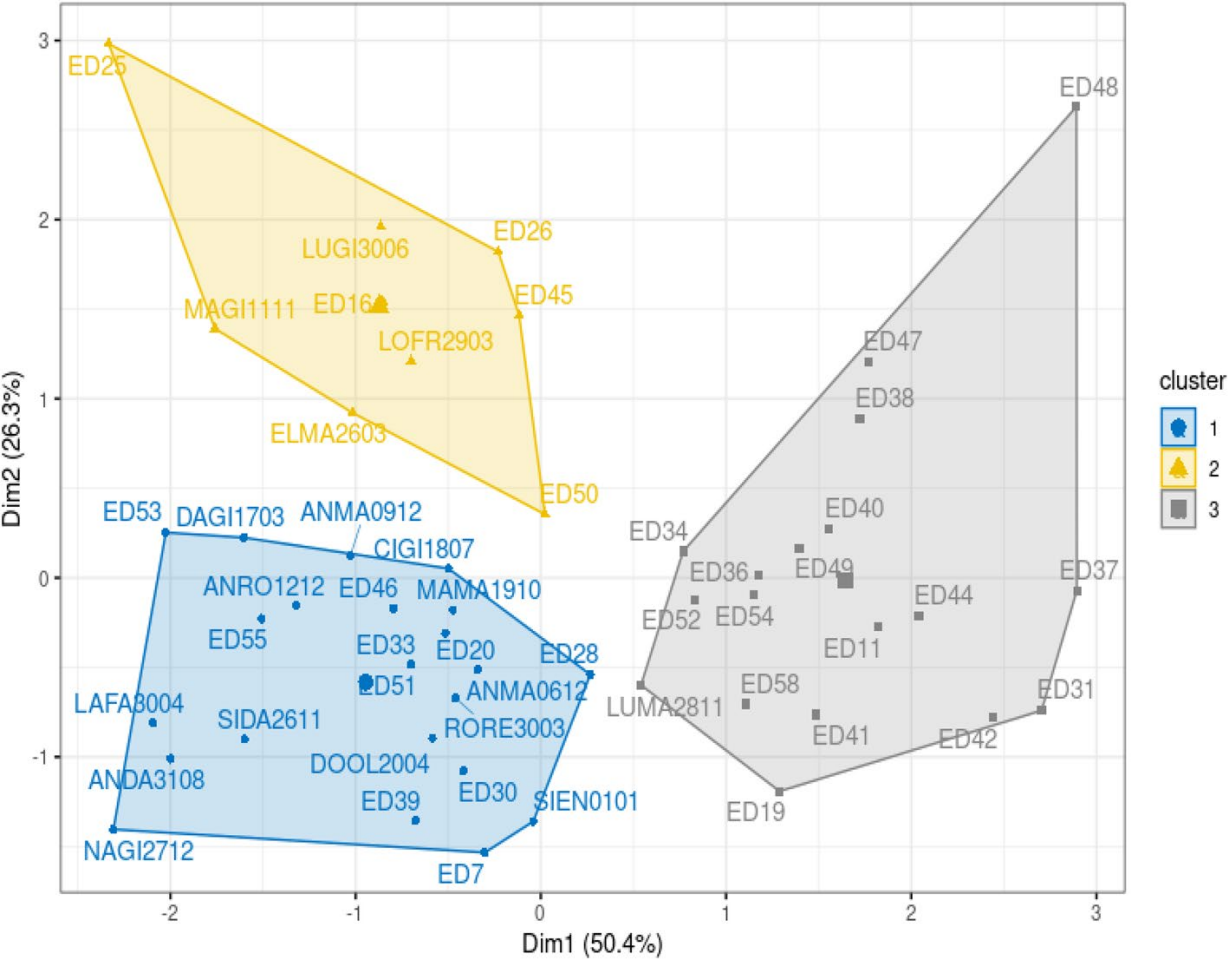
PCA ordination analysis of all samples based on the 4 variables included in the mediation model, and results of HCPC clustering (colour).

To test the mentioned hypothesis, we run a clustering analysis of the samples based on the 4 variables included in the mediation model, namely the CTQ total score, the butyric acid, the STAI trait anxiety, and the EDE-Q total score. PCA analysis with colour and groupings highlighting the identified clusters of samples analysed is reported in Fig. 6. Details of the analysis are reported more in depth explored in Table 6.

**Table 6:** Results of PERMANOVA test to check the association between samples HCPC clustering, and microbial community.

Stratification	Df	F. model	R2	p	BMI factor p	BMI interaction p	Notes
Cluster (1,2,3)	2	1.445	0.058	0.0050	0.0117	0.036	In pairwise comparison, significant difference between cluster 1 and cluster 3

Grouping by cluster is tested against the Bray–Curtis distance matrix of the bacterial community, correcting for BMI (i.e., using and interaction term in the PERMANOVA model).

*BMI* body mass index.

## Discussion

The present study evaluated for the first time the role of both microbiome and SCFAs as mediators of the effects of childhood trauma in a population of patients with EDs. The present investigation was not limited to AN as in most of the previous studies in this field, rather it also included BN and BED. According to the main results, ED patients showed higher overall levels of trauma and anxiety in comparison to HCs. In addition, reduced butyric acid concentration was associated with general (anxiety levels) and specific EDs psychopathology, as well as with childhood traumatic experiences; furthermore, SCFA levels were found to be a mediator of the interactions between these variables, even when adjusting for BMI. Differences in microbial community seem to confirm this trans-nosographic model, being associated with general and specific psychopathology, and with pathological eating behaviours (i.e., binge eating), rather than with specific diagnostic categories.

According to Mack et al.^28^ but in contrast with other observations^39^ SCFAs such as acetic, propionic, and especially butyric acid were not associated with BMI or specific diagnoses, rather with psychopathological variables evaluated in a dimensional approach. Indeed, the lack of association with BMI might suggest that weight fluctuations were unlikely to explain individual differences in SCFAs production, which can be due to more complex behaviours and related modification in colonic SCFA absorption, colonic transit time, and differences in dietary intake^40^.

It is likely that differences between the present study and these previous investigations were due to sample compositions. Indeed, one of the strengths of the present study was to include all the three main EDs diagnostic categories. However, single diagnostic groups considered separately showed reduced dimensions as compared to Borgo et al.^25^ and Mack et al.^28^ studies. Thus, some of the differences previously obtained were not detected, due to the limited sample size.

The HC group can be clearly discriminated from EDs, both when analyzing diversity and richness, and considering PERMANOVA and LEfSe analysis. The analysis allows to re-classify the 3 EDs in 2 groups, based on behavior rather than BMI, binge purging and restricting. In particular the model we developed allows to cluster and clearly discriminate the binge-purging behavior (13 out of the 17 samples in cluster 3. The discriminant taxa associated to the three different ED, described in the LEfSe analysis (Fig. 5) applied to the grouping identifying the well-known SCFAs producers (*Prevotella, Lachnospira, Bacteroides*), and corroborating the power of an analysis integrating microbiome and metabolome.

Nevertheless, as extensively reported, the present study focused on childhood traumatic experiences as a trans-nosographic dimension, and not on diagnostic categories. Indeed, when comparing SCFAs for presence vs absence of trauma, differences were clearly found (especially for butyric acid).

On the other hand, the present result confirmed previous observations regarding the role of butyric acid in the pathogenesis of both depressive and anxiety symptoms^25,41,42^ SCFAs such as butyric acid cross the blood–brain barrier and upregulate several neurotrophic factor expressions in the hippocampus and frontal cortex. Thus, SCFAs were found to further regulate mood^42,43^, modulating serotonergic, and dopaminergic neurotransmission^20,43^.

The model proposed in this study supported the intriguing hypothesis that childhood traumatic experiences might represent a risk factor for gut dysbiosis and for a stable modification of mechanisms responsible for SCFAs production. Considering that both childhood traumatic experiences and onset of eating disorder were evaluated retrospectively, it was not possible to distinguish between alterations specifically due to one of these conditions. However, the results of the present study seem to confirm previous observations, on a complex pattern of interactions between childhood trauma, development of EDs and microbiome alterations. Indeed, the role of childhood trauma in the pathogenesis of EDs has been strongly demonstrated^29^, and it has been suggested that repetitive adverse life events are associated with stable biological alterations, such as hypothalamic–pituitary–adrenal (HPA) axis dysregulation^44^. Early life trauma might challenge microbial colonisation^45,46^, during a critical window of development, and this interference is supposed to impact on the HPA functioning^47,48^. Furthermore, reiterating trauma or stressful conditions during childhood might permanently affect the mutual interaction between the gut microbiota and the brain development^47^. Animal studies seem to support this hypothesis, as for example, premature maternal separation in rats leads to both anxiety and depressive-like behaviours related with significant changes in the composition of the intestinal microbiota^49^. On the other side, alterations in the expression of serotonin transporters following early life stress were responsible for imbalance in the microbial population in rats^50^. Some evidence is also available in humans, as Hemmings et al.^51^ found that history of childhood trauma was associated with lower relative abundance of Actinobacteria and Verrucomicrobia. Furthermore, Flannery et al.^52^ found that *B. fragilis* was inversely correlated with incidents of family turmoil, and it was protective for aggressive behaviour, emotional reactivity, externalising behaviour, sadness, and impulsivity.

The role of SCFAs as potential mediators of the psychopathological consequences of childhood trauma has been suggested, considering that changes in diversity of gut microbiota following early traumatic events might determine reduction of those bacterial species producing butyrate^25,53^. With this regard, Coley et al.^54^ found that history of childhood adversities, measured by the Early Traumatic Inventory Self Report was associated with changes of gut metabolites, including 5-oxoproline, malate, urate, and butyrate, as well as with alterations in functional brain connectivity. The dysbiosis following early adverse events also interacts with other biological systems involved in consequences of trauma such as HPA, as SCFAs and neuropeptides interact with the CNS, activating microglia, which modulate HPA activity^37,55^. On the other hand, neuroinflammation has been postulated to mediate the dysbiosis following early adverse events, as early stress can induce intestinal permeability which contribute to infiltration of B cells and increment of proinflammatory neutrophils and macrophages^37^.

The gut microbiota profiles observed in the present study, partially confirm the suggested model for interaction between childhood trauma and psychopathology in EDs. PERMANOVA analysis was performed to identify potential overlap between clusters of clinical and biological variables with microbiome taxonomy. According to this analysis, different microbiome composition was present for those patients classified in the cluster characterised by childhood trauma, increased anxiety, and binge eating behaviours, as well as reduced butyric acid. In particular, patients reporting binge eating behaviour showed differences in alpha and beta diversity, as well as substantially different bacterial communities compared with both controls and restricting patients, confirming Monteleone et al.^56^, and Mack et al.^28^ findings. At the Genus level, binge-purge patients were characterised by *Prevotella*, while restricting patients by *Bacteroides*, and by the Firmicutes phyla members such as *Facklamia* and *Lachnospira*. In general, a lower bacterial diversity is considered detrimental for (intestinal) health, as it has been linked to different disorders^57^, and it has been associated with both weight fluctuation and general psychopathology^58^. Finally, as a further confirmation to the model, the Bacteroides/Firmicutes ratio was not associated with specific diagnosis, according with Monteleone et al. study^56^, while it was found to be inversely correlated with both anxiety levels and EDs specific psychopathology.

In conclusion, the preliminary findings of the present study suggested a possible association between microbiome taxonomy and a specific cluster of clinical variables, indicating butyrate as a potential mediator of this biological underpinning. Overall, the results appeared to be coherent with clinical observations, as patients with history of childhood trauma have been described to represent a distinct population as compared with other patients with EDs, in terms of more frequent binge-purging behaviours and emotion dysregulation^7,59^. The results of the present study cannot be considered as conclusive, given the methodological limitations. However, they are in line with the need of biological markers of the so-called maltreated echo-phenotype within the EDs population^60,61^. Patients with history of maltreated has been suggested to present also distinct biological profiles^44,62^. A better characterization of these patients might allow evaluating new treatment interventions. Indeed, compared with other psychiatric conditions, EDs do not adequately respond to pharmacological treatments, and despite the innovation of nutrition and psychological interventions, a large proportion of patients still report a chronic long-lasting disorder, with long-lasting and inefficacious treatment. In line with translational medicine, the implementation of models including both psychopathological and biological variables, might allow developing individualised treatments based on specific targets of intervention. Thus, the clarification of the role of microbiome in EDs, might open the way to manipulation of gut microbiota by stimulating a cross-feeding mechanism or even by faecal transplant, and to probiotic supplementation with butyric-like compounds.

The results of the present study should be considered in the light of some limitations. First, the limited sample size might affect the interpretation of the results. In particular, some of the negative results might be due to type II errors. For example, lack of significant difference on CTQ subscales can be due to the small sample size of the single groups. For this reason, the model was run considering CTQ total score.

Then, retrospective and self-reported assessment of childhood traumatic experiences might affect the actual interpretation and the impact of these predisposing factors. Furthermore, the cross-sectional design does not allow establishing causal interactions between variables. Thus, the link between trauma, EDs psychopathology, gut microbiota and its metabolites, and the link between BMI and gut microbiota should be further investigated by means of longitudinal studies. Finally, one of the main limitations of this study is the omission of a group of patients having experienced childhood trauma but having no EDs. Since the main correlations found are between ED-non-specific dimensions, such as trait anxiety, it is possible that these correlations are not ED-specific. Thus, the comparison between ED patients with a history of trauma with ED patients without such a history should be explored in future research.

## Methods

The present study was designed as a cross-sectional investigation, and it was conducted at the Clinic for EDs of the University of Florence. The enrolment took place from January 2018 to February 2022. The study procedures were described to all participants, and signed informed consent was obtained from all participants. The study protocol was approved by the ethics committee of the local institution (Comitato Etico Regione Toscana—Area Vasta Centro), and the study was conducted in accordance with the guidelines of the Declaration of Helsinki of 1964 and subsequent amendments.

### Participants

All subjects taken over for the first time by the clinic were included in our study provided they met these inclusion criteria: female sex; age over 18 years; presence of diagnosis of EDs according to the diagnostic criteria of Diagnostic and Statistical Manual of Mental Disorders—5th ed., text rev. (DSM-5-TR)^3^. The exclusion criteria were as follows: diagnosis of severe psychiatric condition (such as psychosis, major depression disease); intellectual disability; illiteracy, language barrier or any other condition that compromise the understanding of the protocol and the completion of the questionnaires; severe medical condition requiring hospitalisation (e.g., cardiac or renal failure) or which could interfere with microbiome composition such as gastro-intestinal disease (i.e. bowel inflammatory disease or coeliac disease); current use of antipsychotic medication. Of the 69 subjects initially referred, 13 declined to participate and 9 were not included due to exclusion criteria: because of comorbid psychotic disorder (3), severe medical condition (3), below 18 years of age (4). The final sample consisted of 47 patients, aged between 18 and 43 years. A group of healthy subjects also were enrolled as a comparison group among attendants of University Clinic of Florence. The selection criteria for HCs were as follows: female sex; age over 18 years; absence of any psychiatric disorder, based on the diagnostic criteria of the DSM‐5-TR^3^; absence of any previous or current diagnosis of EDs according to the diagnostic criteria of DSM‐5-TR^3^; BMI between 18.5 and 25 kg/m^2^; absence of illiteracy or intellectual disability. Of an initial sample of 31 subjects, 3 were excluded because of psychiatric conditions (depression) (1) and BMI over 25 kg/m^2 (2). The final control sample consisted of 28 subjects.

### Assessment

All patients were recruited on the first day of admission at the clinic. The assessment was carried out by psychiatrists, collecting anthropometric parameters among which weight and height (using standard calibrated instrument) and information regarding potentially traumatic life events, physiological history, pharmacological history. The diagnosis was confirmed with the Structured Clinical Interview for DSM-5 (SCID-5)^63^. For healthy control subjects stool tubes were provided at an appointment at the clinic. Spontaneous stools were collected and stored frozen (− 20 °Cup to 48 h and − 80 °C afterwards) until the processing of faecal samples. They were also given a form with the visual-analogy representation of the Bristol Stool Scale^64^ to classify stools according to shape and consistency and that can be used to monitor the change in intestinal function.

Finally, both patients and healthy controls, were asked to complete these following self-administered questionnaires:

- Eating Disorder Examination Questionnaire (EDE-Q 6.0)^65,66^: a 28‐item test for the assessment of ED‐specific psychopathology. Besides the Total Score, it offers four subscales for the evaluation of the specific components of dietary restraint (EDE‐Q R), eating concern (EDE‐Q EC), shape concern (EDE‐Q SC), and weight concern (EDE‐Q WC).
- SymptomChecklist-90-R—Symptom Checklist-90—Revised (SCL-90-R)^67^: a 90-item questionnaire for the assessment of general psychopathology, which includes a Global Severity Index, obtainable from the average of all items, the Positive Symptom Total (PST), which measure the patient’s tendency to accentuate or minimise symptomatologic discomfort, and the Positive Symptom Distress Index, regarding the number of symptoms reported by the subjects (obtainable from the ratio of the sum of all items and the PST);
- State-Trait Anxiety Inventory (STAI—version Y)^68^: a 40-item test concerning the evaluation of the trait (20 items) and state (20 items) anxiety.
- Beck Depression Inventory (BDI)^69^: a 21-question multiple-choice self-report inventory measuring depression severity.
- Childhood Trauma Questionnaire‐Short Form (CTQ‐SF)^70^: a 28‐item questionnaire which investigates the presence of traumatic experiences during childhood and early adolescence. It provides a Total Score (CTQ TS) and five subscales, for assessing the subject's exposure to specific categories of maltreatment: emotional neglect (CTQ EN), emotional abuse (CTQ EA), physical neglect (CTQ PN), physical abuse (CTQ PA), and sexual abuse (CTQ SA). A cutoff value of 35 is commonly used to identify the presence of significant early trauma.
- Emotional Eating Scale (EES)^71^: a 25-item scale which offers three subscales (EES Anger/Frustration, EES Anxiety, EES Depression) beside the Total Score, investigating the relationships between specific negative emotional states and overeating.

### Dietary information of patients

All patients underwent a structured nutritional rehabilitation, with intervention strategies aimed at the progressive reorganization of the mechanisms of regulation of appetite and body weight. The dietetic-nutritional path was based on a transdiagnostic perspective focused on a personalized dietary-nutritional path in relation to the estimated needs for each person. Specifically, in patients with AN, nutritional rehabilitation consisted of a three steps oral intake, with regular increase of 500 kcal (1st step 1000 kcal/59 g protein/34 g lipids/123 g carbohydrates—2nd step 1500 kcal/77 g protein/33 g lipids/212 g carbohydrates -3rd step 2000 kcal/96 g proteins/62glipids/320 g carbohydrates). Patients with BN and BED follow essentially the same path: meal assisted with a caloric-nutritional adaptation in relation to the different nutritional needs. The proposed diet will therefore be normocaloric, sometimes hypercaloric with a macronutrient intake of 10–15% proteins, 20–35% lipids; 45–60% glycides starting from estimation of the patient's caloric intake aimed not exclusively at weight management bodily but to the management of dysfunctional behaviors.

### Faecal water preparation and SCFAs and MCFAs quantification

Each collected sample was thawed and weighted (weight range 500–800 mg), then added sodium bicarbonate 10 mM (1:1 w/v) in a 1.5 mL centrifuge tube. The obtained suspension was then mixed with the aid of a sterile wooden stick and briefly shaken in a vortex apparatus, extracted in ultrasonic bath (15 min) and then centrifuged at 4 °C at 13.000 *rpm* for 90 min. The supernatant was collected, transferred in 1.5 mL centrifuge tube and stored at − 20 °C until use. For the analysis, these supernatant samples were thawed, briefly centrifuged at 5000 *rpm* and resuspended for 5 min in an ultrasonic bath. The SCFAs and MCFAs were then extracted as follows: an aliquot of 100 μL of sample solution (corresponding to 0.1 mg of stool sample) was added with 10 μL of internal standard (ISTD) mixture, 1 mL of tert-butyl-methyl ether and 50 μL of 1.0 M HCl solution in 1.5 mL centrifuge tube. Afterwards, each tube was shaken in a vortex apparatus for 2 min, centrifuged at 10,000 *rpm* for 5 min; the solvent layer was finally transferred in auto-sampler vial and analysed by Gas-Chromatography–Mass Spectrometry (GC–MS) method, using an Agilent GC–MS system composed with 5971 single quadrupole mass spectrometer, 5890 gas-chromatograph and 7673 autosampler^72^.

### Bacterial DNA extraction, 16S rRNA gene sequencing, and sequencing data analysis

Bacterial DNA extraction, sequencing, and sequencing data analysis were performed as previously described^72,73^. Briefly, DNA extraction was performed with the DNeasy PowerLyzer PowerSoil Kit (Qiagen, Hilden, Germany). Library preparation and sequencing of the hypervariable region V3–V4 of the 16S rRNA gene were performed by using an Illumina MiSeq platform with a 300-bp paired-end reads protocol. The obtained reads were pre-processed with CUTADAPT^74^ to remove primers and Illumina adapters, while SICKLE was used to remove low quality portions of the reads^75^. OTUs/ASVs identification was performed in MICCA (ver. 1.7.2)^76^ with miccaotu command, using the UNOISE3 algorithm^77^, while taxonomy was assigned using the RDP classifier (Ver 2.11)^78^ against the RDP database.

The datasets generated during and/or analysed during the current study are available in the ENA repository, under accession number PRJEB55035.

### Statistical analyses

Clinical, biological and psychometric continuous measurements were reported using means and standard deviations and compared between diagnostic groups using BMI-adjusted Analysis of Covariance (ANCOVA). Statistically significant comparisons were further investigated through post-hoc analyses using the Tukey method for multiple corrections.

Associations between fatty acid concentrations and psychopathological variables were tested using multiple linear regression models, with each fatty acid entered individually as an independent variable and BMI as a covariate.

The proposed serial mediation model was tested through mediation analysis, with CTQ Total Score as independent variable, EDE-Q Total Score as dependent variable, and butyric acid and STAI Trait Anxiety as mediators in series. For all three possible indirect effects (two single-mediator effects and one with both mediators in series), 95% confidence intervals were computed using bias-corrected bootstrapping with 10,000 resamples; indirect effects were considered statistically significant when their confidence intervals did not include zero. BMI was entered as a covariate in all regression equations. Both unstandardized and standardised coefficients were computed and reported. All analyses were computed using R Statistical Software version 4.1.2, and the following packages: dplyr, lavaan^79^.

### PERMANOVA analysis

Statistical analysis of the gut microbiota data was performed using R Software similarly to how it was previously described^72^. Sequencing count data (i.e., the ASV table and ASV taxonomy) were parsed into R using phyloseq^80^, and prior to further analysis, reads counts were transformed with CSS (Cumulative Sum Scaling) followed by logarithm transformation as implemented in metagenomeSeq package^81^.

Hierarchical Clustering on Principal Components (HCPC) analysis in the FactoMineR package^82^ was used to assess if patients could be divided in data-driven clusters using the variables from the psychopathology mediation model (4 variables included in the mediation model: namely the CTQ total score, the butyric acid, the STAI trait anxiety, and finally the EDE-Q total score). In HCPC, dimensionality of the data (which in this case is 4) is first reduced with the aid of principal component analysis, and then hierarchical clustering is applied. One of the objectives was to finally test if those data-driven clusters had (if any) a better relation to the gut microbiota with respect to other possible classifications.

We performed different a-priori subjects stratifications based on the above-mentioned clusters. The relation between those different stratifications, and the faecal microbiota community structure, were tested using PERMANOVA with BMI as the controlling factor. Permutational multivariate analysis of variance (PERMANOVA, 9999 permutations, Bray–Curtis dissimilarity), as a non-parametric and permutational method for multivariate analysis of variance, is a commonly used method in the gut microbiota data analysis field. The objective is to test if the centroids and dispersion of the groups in the multivariate space are equivalent for all groups (i.e. the null hypothesis).

Alpha diversity in bacterial communities was explored using three indices: Species Richness (i.e. the number of different OTUs in a sample), Evenness index (i.e. the grade of equitability in the distribution of relative abundances of the OTUs in a sample), and Shannon index (i.e. a measure of diversity of the community in a sample). Differences in alpha diversity indices between samples assessed with the Wilcoxon test.

Beta-diversity of bacterial communities was explored and visualised using Principal Coordinate Analysis (PCoA) ordinations based on the Bray–Curtis dissimilarity, with the phyloseq package. Associations of bacterial community diversity with the main variables resulting from the psychopathology mediation model (butyric acid, STAI trait, CTQ total, and EDE total) were assessed using PERMANOVA with BMI as correcting interaction. Significant relations were further visualised as colour of points on the PCoA ordination.

LEfSe (Linear discriminant analysis Effect Size) analysis^83^ on the CSS transformed abundances, was performed using an online Galaxy implementation^84^ to identify plausible bacterial biomarker(s) able to separate different groups (i.e. according to different patient classification, and with respect to healthy control). Significance threshold for Kruskal–Wallis rank-sum test was 0.05, and threshold for LDA (linear discriminant analysis) was 2.

## Data Availability

The datasets used and/or analyzed during the current study are available from the corresponding author on reasonable request.
